# Factors associated with child delivery expenditure during the transition to the national implementation of the two-child policy in China

**DOI:** 10.1186/s12955-021-01678-z

**Published:** 2021-01-22

**Authors:** Shuang Zang, Jing OuYang, Meizhen Zhao, Yalan Zhu, Jia Liu, Xin Wang

**Affiliations:** 1 School of Nursing, China Medical University, Shenyang, China; 2 College of the Humanities and Social Sciences, China Medical University, Shenyang, 110122 Liaoning China; 3 Humanity and Management College, Shaanxi University of Chinese Medicine, Xianyang, China; 4 Nursing Department, Tongji Hospital Affiliated to Tongji Medical College, Huazhong University of Science and Technology, Wuhan, China

**Keywords:** Health expenditure, Family planning, Child delivery, Birth cost, Two-child policy, China

## Abstract

**Background:**

This study aimed to analyze the status of birthrates and the characteristics of child delivery expenditure under the Chinese two-child policy’s transition period. We evaluated the socioeconomic factors associated with child delivery and provide evidence for decisions relating to health support for childbirth.

**Methods:**

Child delivery expense data were obtained from 2015 to 2017 in Dalian, China. A total of 13,535 obstetric records were enrolled using stratified random sampling and the proportional probability to size method. First, we calculated the current curative expenditure of child delivery and health financing in childbirth costs based on the System of Health Accounts 2011 (SHA 2011). Second, univariate analysis of variance and generalized linear modeling were performed to examine factors associated with child delivery expenditure. Third, we classified the included hospitals into the county, district, and municipal hospitals and compared maternal characteristics between these categories.

**Results:**

Overall, out-of-pocket payments accounted for more than 35% of the total expenditure on child delivery. Median (interquartile range) delivery expenditure at the county and district level hospitals [county-level: 5128.50 (3311.75–5769.00) CNY; district-level: 4064.00 (2824.00–6599.00) CNY] was higher than that at the municipal level hospitals: 3824.50 (2096.50–5908.00) CNY. The increase of child delivery expenditure was associated with an increased ratio of reimbursement, admissions to county and district level hospitals, cesarean sections, and length of stay, as well as a decline in average maternal age (*p* < 0.05).

**Conclusions:**

Health financing for childbirth expenditure was not rational during the transition period of the family planning policy in China. Higher delivery expenditure at county and district level hospitals may indicate variations in medical professionalism. Poorly managed hospitalization expenditure and/or nonstandard medical charges for childbirth, all of which may require the development of appropriate public health policies to regulate such emerging phenomena.

## Introduction

Low birth rates, an imbalanced sex ratio, and an aging society are issues that have plagued many countries [1, 2]. Influenced by the transformation of family planning policy, fertility willingness, and social-economic system, China's population and family structure have changed dramatically over the past 3 decades (e.g., the increasing proportion of the empty nest family and no kids (DINK) family) [3]. In early 2016, China responded to these issues by updating its family planning policy to allow citizens to have up to two children. Contrary to expectation, this policy change was not followed by a significant increase in the birth rate. According to the annual Statistical Bulletin on National Economic and Social Development, despite a brief increase in 2016, the number of newborn babies in China fell considerably in the following years: with 16.87 million in 2015 [4], 17.86 million in 2016 [5], 17.23 million in 2017 [6], and finally 15.23 million in 2018 [7]. Liaoning province has been experiencing a negative year-on natural population growth rate since 2015, and in particular, its birth rate (6.49‰) was only half of the national birth rate (12.43‰) in 2017 [8]. During 2015 and 2016, this province became one of the three representative provinces with “lowest birth rate, moderate mortality rate, lowest population growth rate” in China [9]. The population problem in Liaoning province needs to be addressed. The most common explanations for this trend are the high cost of living, housing, education, and the ideational changes about having children among young couples [10]. Liaoning’s overall economic level is intermediate in the country, and in 2018, it ranked 13th among the 31 provinces on the Chinese mainland in terms of GDP per capita [11]. Previous research has indicated that the fertility decline is partly due to social and family economic conditions in local areas [12]. A birth intention survey showed that women with more economic and social resources were more likely to have a second child [13]. However, another study indicated that income was not a deciding factor for planning to have a child [14]. The findings of the relationship between fertility ideation and economic aspects were mixed in the existing literature. More study is needed to analyze the economic factors associated with the low-fertility trap in the context of China’s two-child policy.

Given that the cost of childbirth is a significant part of the child-rearing expenditure and an essential medical event in the lives of most women, it may also play an important role in childbirth ideation. The World Health Organization indicated that the amount spent on health was related to individuals’ current and expected levels of fitness [15]. Delivery cost should also be considered in conjunction with the quality and outcomes of health care, as lower prices may not reflect high-value care [16]. Compared to per capita disposable income, the costs incurred by delivery are not very high in China, and the expenditure of giving birth may not bring substantial family economic burden [17]. However, among the top four factors considered by 80% of parents to influence the childbirth were: fertility cost, economic burden, care burden, and public service factors [18]. A study in 2016 also showed that the medical payment method (i.e., insurance, out-of-pocket) was the most significant influencing factor for mothers who were planning to have a second child [19]. Delivery expenditure and its potential socioeconomic factors concern people's livelihood. Few studies have explored the associated factors of delivery expenditure or discussed the sociological factors behind delivery expenditure during China’s policy transition. This study is set in Dalian, Liaoning province, where fertility rates are low just as throughout the province. We aimed to explore the expenditure of child delivery during the policy change era and analyze factors associated with fertility.

In this project, we collected information on the delivery expenditure of childbearing women and used the System of Health Account 2011 (SHA 2011) as methodological guidance to calculate the current curative expenditure. SHA 2011 is considered a global criterion for the construction of national health accounts formulated by the Organization for Economic Cooperation and Development, the World Health Organization, Eurostat, and the European Union [20]. Formulating health expenditure under SHA 2011 allows a more accurate calculation of health costs, as it can separate current expenditure from the gross capital formation in health care [21]. SHA 2011 defines the current curative expenditure (CCE) as the final consumption expenditure of resident units on health care goods and services, which does not include the fixed assets’ cost and contributes to calculating the direct health expense [22]. Notably, this study put emphasis on the CCE of child delivery and expected to explore more accurately the socioeconomic factors relating to China’s fertility problems.

## Methods

### Data sources

The dataset on child delivery expenditure in Dalian consisted of macro data (economics, health and demographic information) extracted at the provincial and municipal levels from annual working documents, specifically, from Liaoning Health Statistical Yearbook (2015–2017), Liaoning Health Financial Yearbook (2015–2017), Dalian Health Report (2015–2017), as well as sample institutions’ medical records. Permission was granted by the health commission of Liaoning province and Dalian city for data extraction.

### Sample method and sample size

This project utilized stratified random sampling and the probability proportional to size method to select representative hospitals, using the geographical (urban versus rural area) and administrative (e.g., street-based) location as strata. One-third of the sample was randomly selected from each stratum using a computer-generated random sequence (EPIDATA 3.1 software). The sampling process adopted a step-by-step method. In the first step, one-third of the municipal-level hospitals in the city were taken as a sample. In the second step, district-level hospitals and county-level hospitals were selected at random using the probability proportional to size method. In the third step, districts of the urban city and rural counties were selected by stratified random sampling. Fourth, five to eight streets were selected in of the previously selected counties and districts, and four to six communities were then chosen from the administrative area surrounding each street. For rural counties, twenty towns were initially sampled, and then two to six villages were randomly picked from each town using the probability proportional to size method. In the fifth step, medical institutions were randomly selected from the areas chosen in the fourth step. The number of sampled institutions was different in each of the 3 years due to the application of the probability proportional to size method. In the first sampling year (2015), to ensure a broad health institution inclusion, the institutions were selected by expanding the calculated decimal numbers up to the nearest higher integers in each step (for instance, if 3.3 was the calculated number, 4 institutions would be selected). After the first sampling year, sampling was done with the calculated numbers rounded to the nearest integers in each step in the study year of 2016 and 2017 (for instance, 3.3 rounded down to 3, while 3.6 rounded up to 4). As a result, 565, 496, and 456 medical institutions were randomly sampled from Dalian in 2015, 2016, and 2017, respectively. Most of the sample medical institutions do not provide delivery services; only twelve hospitals (2015), seven hospitals (2016), and seven hospitals (2017) were the source of data on delivery in this study.

For sampled hospitals, the cluster sampling method was used to select maternal samples. We selected the childbearing women whose medical records were coded O80 to O84 according to the tenth revision of the International Classification of Diseases [23]. To ensure the quality of the data, researchers checked and standardized the information that was uploaded by the sample institutions. The records with incomplete and/or unclear information were returned to the source institutions for correction and resubmitted. At the analysis stage, records with abnormal or missing indicators were eliminated from this study (3.10% of the total sample). The child delivery expenditure includes payment for the hospital stay, drug/medication expenses, laboratory expenses, pay for cesarean sections, nursing expenses, spending on blood, and other related costs. We also collected some information, including age, insurance status, delivery mode, length of stay, hospital level, hospital type, and hospital location. Finally, a standardized database was created with 13,535 eligible records in this study. In total, 8004, 2354, 3177 records were included in the year of 2015 to 2017, respectively.

### Statistical analysis

The annual delivery cost was calculated as the sum of hospital charges from admission to discharge for each patient, converted to CCE under the framework of SHA 2011. The formula used to calculate the CCE was derived from Duan et al. [24]. According to the Kolmogorov–Smirnov test, the delivery expenditure, length of stay, and maternal age were not normally distributed. Ordinal variables were reported using descriptive statistics (median and interquartile range). Kruskal–Wallis tests and Wilcoxon rank-sum tests were conducted to assess between-group comparisons. Because the delivery expenditure data were overly dispersed, the generalized linear model with negative binomial distributions was used for multivariate analysis to evaluate the effects of categorical and numerical variables on delivery expenditure. The level of statistical significance was set at *p* < 0.05. Data were analyzed using SPSS 21.0 (SPSS Inc., Chicago, IL, USA) and Stata 15.0 (Stata Corp, College Station, TX).

## Results

### The situation of childbirth in Dalian from 2015 to 2017

Descriptive information about annual births and financing is displayed in Table 1. The birth rate rose from 8.93‰ in 2015, to 9.44‰ in 2016, to 9.60‰ in 2017. However, the number of annual births rose considerably after the implementation of the two-child policy from 45,714 in 2015 to 69,544 in 2016, before falling somewhat to 60,265 in 2017. The financing for child delivery was from three major sources: public financing scheme, voluntary financing scheme, and out-of-pocket payments (OOPs). During the three study years, the ratio of OOPs/CCE remained at a relatively high range, with a low of 36.71% in 2016 to a high of 37.99% in 2017. The rate of government financing scheme was less than 13% in all years (Table 1). There were 10,358 mothers age ≥ 35 years, which accounted for 17.36% of the number of women giving birth in Dalian, 2017—this was 19.82% higher than the same number in 2016. Conversion rates in 2015, 2016, and 2017 were 1 USD ≈ 6.23 CNY, 6.64 CNY, and 6.75 CNY, respectively.

**Table 1:** Allocation of the birth and the financing scheme for delivery (million CNY)

Year	Newborns (n)	(Crude) birth rate (‰)	Public financing scheme		Voluntary financing scheme		OOPs	Delivery CCE	Ratio of OOPs/CCE (%)
			Social health insurance	Government financing scheme	Voluntary health insurance program	Enterprise financing plan			
2015	45,714	8.93	42.69	10.56	3.58	7.15	37.75	101.73	37.11
2016	69,544	9.44	57.14	12.44	4.34	9.47	48.37	131.76	36.71
2017	60,265	9.60	53.32	10.77	4.23	8.10	46.82	123.24	37.99

### Factors associated with delivery expenditure by univariate analysis

We used the univariate analysis to identify factors associated with the child delivery expenditure. The sampling scheme allowed more institutions to be sampled in 2015 than in 2016 and 2017. Sampling bias were aggravated by the fact that the number of yearly births in each institution was heavily skewed, ranging from 0 to thousands. With more institutions sampled in 2015, more maternal records were harvested than in 2016 and 2017. Due to the unevenness and skewedness in the numbers of maternal records in the 3 years, comparison of delivery expenditure among the study years was waived. Table 2 provides the descriptive statistics of the explanatory variables. There were substantial differences in the median (and interquartile range) delivery costs per hospitalization, depending on the delivery mode, insurance status, institution level, and hospital type. Compared to high-level hospitals, the delivery costs for childbearing women were significantly higher in county and district level hospitals (*p* < 0.001). There were no statistically significant differences in delivery expenditure between rural and urban hospitals (*p* = 0.401).

**Table 2:** Median child delivery expenditure by delivery mode, insurance status, and hospital characteristic (n = 13,535)

Variables	n	Delivery expenditure Median (P_25_ to P_75_)	*H*/*Z*	*p* value	Post hoc
Delivery mode			− 19.053	< 0.001	–
Vaginal delivery	12,826	4070.00 (2771.00 to 5885.00)			
Cesarean section	709	6324.50 (5222.00 to 7911.00)			
Insurance status			300.718	< 0.001	IS2 > IS1(*p* < 0.001); IS3 > IS1(*p* < 0.001); IS4 > IS1(*p* < 0.001); IS5 > IS1(*p* < 0.001); IS3 > IS2(*p* = 0.001); IS4 > IS2(*p* < 0.001); IS5 > IS2(*p* < 0.001)
Self-funded	9052	3978.00 (2493.00 to 6049.00)			
New rural cooperative medical scheme	3671	4498.00 (3252.00 to 5757.00)			
Urban and rural medical insurance	39	6090.00 (3297.00 to 7840.00)			
Urban residents’ basic medical insurance	16	6080.00 (4738.50 to 7229.25)			
Urban employees’ basic medical insurance	557	6001.00(3307.00 to 10,658.50)			
Hospital level			241.336	< 0.001	HL2 > HL1(*p* < 0.001); HL3 > HL1(*p* < 0.001); HL3 > HL2(*p* = 0.037)
Municipal	5670	3824.50 (2096.50 to 5908.00)			
District	4706	4064.00 (2824.00 to 6599.00)			
County	3159	5128.50 (3311.75 to 5769.00)			
Hospital type			355.484	< 0.001	HT2 > HT1(*p* = 0.003); HT3 > HT1(*p* < 0.001); HT3 > HT2(*p* < 0.001);
Traditional Chinese medicine hospital	1189	3481.00 (2647.00 to 5510.50)			
Maternal and childcare hospital	6877	4278.00 (2597.50 to 5750.50)			
General hospital	5469	4306.00 (3059.50 to 8029.50)			
Hospital location			− 0.840	0.401	
Urban	6697	4370.50 (2861.50 to 5982.75)			
Rural	6838	4100.50 (2777.00 to 6089.25)			

For the reimbursement ratio, urban employees’ basic medical insurance > urban residents’ basic medical insurance > urban and rural medical insurance > new rural cooperative medical scheme

*IS* insurance status, *IS1* self-funded, *IS2* new rural cooperative medical care, *IS3* urban and rural medical insurance, *IS4* urban residents’ basic medical insurance, *IS5* urban employees’ basic medical insurance, *HL* hospital level, *HL1* municipal hospital, *HL2* district hospital, *HL3* county hospital, *HT* hospital type, *HT1* traditional Chinese medicine hospital, *HT2* maternal and childcare hospital, *HT3* general hospital

### Factors associated with delivery expenditure by multivariate analyses

As the negative binomial regression model better accommodates data with over-dispersion, so we used a generalized linear model (with negative binomial distributions) to explore the factors associated with the child delivery expenditure. The value of Deviance/*df* and Pearson chi-square/*df* were 0.519 and 0.565, respectively. These were closer to 1.0 than to 0.0 and were greater than 0.05, so the model fit statistics were good. Table 3 presents the econometric results of the general linear model for childbearing women’s expenditure of hospitalization. Cesarean sections, maternal age under 35, district and county level hospital, high reimbursement ratio, and long-term hospitalization increased child delivery fees significantly (*p* < 0.001).

**Table 3:** General linear model with child delivery expenditure as the dependent variable

Parameters	*β*	*p* value	Exp(*β*)	95% Wald confidence interval for Exp(*β*)	
				Lower	Higher
(Intercept)	9.251	< 0.001	10,409.831	8888.787	12,191.154
Delivery mode					
Vaginal delivery	− 0.214	< 0.001	0.808	0.747	0.873
Cesarean section^a^	0^b^	–	1	–	–
Insurance status					
Self-funded	− 0.414	< 0.001	0.661	0.607	0.721
New rural cooperative medical scheme	− 0.552	< 0.001	0.593	0.540	0.652
Urban and rural medical insurance	− 0.218	0.026	0.804	0.664	0.974
Urban residents’ basic medical insurance	− 0.293	0.004	0.746	0.610	0.912
Urban employees’ basic medical insurance ^a^	0^b^	–	1	–	–
Hospital level					
Municipal hospital	− 0.145	< 0.001	0.865	0.821	0.911
District hospital	0.191	< 0.001	1.211	1.150	1.275
Country hospital^a^	0^b^	–	1	–	–
Maternal age	− 0.015	< 0.001	0.986	0.983	0.988
Length of stay	0.030	< 0.001	1.056	1.050	1.063

^a^Reference value

^b^This parameter is set to zero because it is redundant

### Maternal characteristics in different levels of hospitals

Table 4 shows that childbearing women who gave birth in the district and county level hospitals stayed longer than those who gave birth in high-level hospitals (*H* = 855.777, *p* < 0.001). Mothers with low reimbursement rates tended to choose district and county hospitals for delivery. In contrast, mothers who were self-funded and who had urban residents’ basic medical insurance tended to give birth in municipal hospitals. Meanwhile, mothers with insurance status of new rural cooperative medical scheme and urban and rural medical insurance were more likely to give birth in county hospitals.

**Table 4:** Maternal characteristics in different levels of hospitals

Variables	Municipal hospital	District hospital	County hospital	*H*	*p* value
	Median (P_25 _− P_75_)/n(%) [Adjusted residual]				
Length of stay (day)	4 (3–6)	5 (4–6)	6 (4–9)	855.777	< 0.001
Maternal age (year)	29 (27–33)	29 (27–34)	29 (27–35)	45.438	< 0.001
Insurance status (n)				4039.082	< 0.001
Self-funded	4918 (86.7) [41.7]	3113 (66.1) [− 1.3]	1021 (32.3) [− 47.1]		
New rural cooperative medical scheme	429 (7.6) [− 43.5]	1241 (26.4) [− 1.5]	2002 (63.4) [52.3]		
Urban and rural medical insurance	0 (0.0) [− 10.1]	3 (0.1) [− 8.1]	136 (4.3) [20.9]		
Urban residents’ basic medical insurance	2 (0.0) [− 8.8]	114 (2.4) [14.4]	0 (0.0) [− 6.0]		
Urban employees’ basic medical insurance	321 (5.7) [7.7]	235 (5.0) [3.8]	0 (0.0) [− 13.3]		

## Discussion

The implementation of the two-child policy ignited a heated national debate regarding the birth rate as well as the complexity of parenting and the social impact of this policy [25]. This study found that after the 2016 implementation of the new birth policy, there was a surge in the number of newborns in 2016. However, the growth in the number of newborns did not maintain momentum in Dalian in 2017. Although there was a slight increase in birth rate in 2017, the number of newborns did not increase. This was probably due to population outflow (e.g., migrant workers, retirement in different places) [26], which produced a temporary effect on birth rate. A recent study on married professional females in Dalian showed that the women were reluctant to have a second child [27]. Childbirth ideation and parenting desires are not strongly impacted by government policy, but rather by the careful consideration of people’s long-term interests and personal goals [28].

In November 2015, an estimate found that more than 90 million couples in China were eligible to have a second birth according to the two-child policy, of which, 60% were more than 35 years old [29]. Our study echoed this finding. The two-child policy did lead to a boom of pregnancies among women aged greater than 35. This is due to the two-child policy providing legal permission for those previously ineligible to have a second child [30]. At the same time, for women with advanced age, the time window that favors pregnancy is narrowing.

Over the study period, the ratio of OOPs/CCE kept at a relatively high amount above 37%, while the rate of government financing scheme was less than 13%. Given that childbirth is of societal importance in China (as it is in all countries), the associated costs ought to be borne by the whole of society [31]. To encourage childbearing, in recent years, China introduced a series of methods to subsidize childbirth, including increasing the reimbursement for maternity insurance [32]. However, like in most other developing countries, there is a regional difference in financial access to maternal health within China [33]. Local birth subsidy policy is uneven, and there exists a disparity in health insurance and the distribution of health care financing [34]. Maternity insurance is one of the major sources of regional disparity in delivery expenditure. The implementation programs for maternity insurance are diverse in different cities [35]. In this study, women in Dalian did not benefit much from the maternity insurance schemes, which is substantiated by the fact that as high as 66.88% of the sampled women had to pay the delivery fee out of pocket.

This study also found that residents with lower medical insurance reimbursement rates tended to choose district and county hospitals to give birth instead of municipal hospitals, which may be attributed to concerns about the high medical costs that may accompany high-level hospitals. Therefore, under such circumstances, the perceived fertility cost pressure may become a critical issue to reduce fertility desire and thus inhibit the effect of the two-child policy [36]. At the same time, this is also providing evidence of the uneven distribution of high-level hospitals in the city. Delivery is sometimes an urgent medical matter, and the choice of maternal delivery medical institutions is often restricted by the distance from the home to the hospital [37]. Therefore, most rural women tend to give birth in district or county hospitals near their homes.

In China, each province and city has its health care system, mostly influenced by regional economic factors [38]. Dalian is one of the 15 sub-provincial cities and 14 coastal open cities in China. With the acceleration of economic development and urbanization over the past decades, Dalian keeps expanding, and many rural or peri-urban areas are becoming urban. At the end of 2017, the population urbanization rate was 72% in Dalian [39]. Accordingly, there were no significant differences in delivery expenditure between urban and rural hospitals in this study.

In addition to calling on the government to subsidize birth, work can be done on the factors associated with the health expenditure on delivery. As we can get from the results, high reimbursement ratio, district and county hospital, cesarean sections, and extended length of stay were associated with higher delivery expenditure. The results were in line with a previous study in India, where the place of birth, type of delivery, and financial subsidy for childbearing were influencing factors of spending related to delivery [40]. Additionally, the length of stay is a crucial indicator of medical resource consumption in obstetric delivery [41], providing the alternative explanation that costs rise with longer stays in the hospital. Meanwhile, it seems possible that patients with severe health issues spend a long time in hospital and depend more on health resources.

Among the factors we included, maternal age significantly associated with delivery expenditure—the lower the childbearing age, the higher the delivery cost. One possibility is that most of the older women in our sample were having their second children, smoothing the delivery process. Furthermore, the experience with the first birth can shape subsequent fertility-related decisions, causing selection bias where older women are more likely to choose to have a second child if their first delivery went well [42].

In this study, county and district level hospitals appear to charge childbearing women more significantly in comparison to municipal hospitals, and we identify several possible explanations. Further exploration revealed that the percentage of uninsured mothers giving birth in high-level hospitals were higher than those in low-level ones. This was consistent with previous research, which found that the proportion of uninsured persons was higher in urban hospitals than that in rural medical institutions [43]. As insurance status was a significant variable associated with delivery expenses, we speculated that the association between high-level hospitals and lower charges might be related to a higher proportion of uninsured childbearing women that they served. From this study, women stayed longer in low-level hospitals than those in high-level hospitals, which may also be a reason for the spending differentiation. Variations in clinical decision making may also occur owing to the limited availability of experts in low-level hospitals, which potentially contributing to higher expenditure [44]. Meanwhile, the etiology of disparity in different levels and types of hospitals’ delivery costs may be attributed to a metropolitan-specific normative sanitary management system [45]. That is, the combination of variations in medical decisions and differences in hospital charges could drive differences in health expenditure when caring for the roughly homogeneous group of childbearing women. Further research on the impact of hospital's characteristic on delivery expenditure of puerpera is recommended.

## Strengths and limitations

The Strengths of this study include data of delivery expenditure during the period of policy change, which enabled us to know the CCE of delivery and health financing characteristics related to childbirth in China in this critical period. This study adds to evidence for the health economy from the perspective of delivery expenditure and supplements the findings available for socioeconomic factors in China related to childbirth. Several limitations were also present. First, the cross-sectional property and the “hit and miss” aspect generated from a stratified random sampling of medical institutions may induce some selection biases. Second, as this was an analysis of health report data, the availability variables were limited. Factors introduced in this study were part of socioeconomic indicators associated with the delivery expenditure, and future research may investigate other possible related factors.

## Conclusions

This study examined trends in the delivery expenditure as well as the factors associated with it. The results have implications for the future development of population dynamics. After implementing the two-child policy, the number of newborns was higher than before the policy was introduced but showed a downward trend. The adjustment of the family planning policy alone could not keep on contributing to the demographic. Our findings confirm fertility issues should be considered within the socioeconomic context of the population. The results suggest an unreasonable proportion of health financing, and different levels of hospitals are associated with disparity delivery expenditure. These findings deserve attention and in-depth study. Some results of this study may contribute to the currently undergoing healthcare reform during the birth policy adjustment period.

## Data Availability

All data sets supporting the conclusions of this article are included in the article.

## Abbreviations

CCE: Current curative expenditure
SHA 2011: System of Health Accounts 2011
OOPs: Out-of-pocket payments
